# Migration Challenges and Their Impact on the Primary Healthcare System—A Qualitative Research

**DOI:** 10.3390/healthcare12161607

**Published:** 2024-08-12

**Authors:** Olga Partyka, Monika Pajewska, Aleksandra Czerw, Katarzyna Sygit, Oleh Lyubinets, Tomasz Banaś, Krzysztof Małecki, Elżbieta Grochans, Szymon Grochans, Anna Cybulska, Daria Schneider-Matyka, Elżbieta Cipora, Mateusz Kaczmarski, Krzysztof Sośnicki, Grażyna Dykowska, Zofia Sienkiewicz, Łukasz Strzępek, Ewa Bandurska, Weronika Ciećko, Jarosław Drobnik, Piotr Pobrotyn, Aleksandra Sierocka, Michał Marczak, Remigiusz Kozlowski

**Affiliations:** 1 Department of Health Economics and Medical Law, Medical University of Warsaw, 01-445 Warsaw, Poland; 2 Department of Economic and System Analyses, National Institute of Public Health NIH—National Research Institute, 00-791 Warsaw, Poland; 3 Faculty of Health Sciences, Calisia University, 62-800 Kalisz, Poland; 4 Department of Public Health, Danylo Halytsky Lviv National Medical University, 79010 Lviv, Ukraine; 5 Department of Radiotherapy, Maria Sklodowska-Curie Institute-Oncology Center, 31-115 Cracow, Poland; 6 Department of Radiotherapy for Children and Adults, University Children’s Hospital of Cracow, 30-663 Cracow, Poland; 7 Department of Nursing, Faculty of Health Sciences, Pomeranian Medical University in Szczecin, 71-210 Szczecin, Poland; 8 Department of Pediatric and Oncological Surgery, Urology and Hand Surgery, Faculty of Medicine and Dentistry, Pomeranian Medical University in Szczecin, 71-252 Szczecin, Poland; 9 Medical Institute, Jan Grodek State University in Sanok, 38-500 Sanok, Poland; 10 Department of Nursing, Social and Medical Development, Medical University of Warsaw, 02-091 Warsaw, Poland; 11 Department of Surgery, Andrzej Frycz Modrzewski Cracow University, 30-705 Cracow, Poland; 12 Center for Competence Development, Integrated Care and e-Health, Medical University of Gdansk, 80-204 Gdansk, Poland; 13 Department of Family Medicine, Faculty of Medicine, Wroclaw Medical University, 51-141 Wroclaw, Poland; 14 Pulsantis Specialist and Rehabilitation Clinic Ltd., 53-238 Wroclaw, Poland; 15 Department of Management and Logistics in Healthcare, Medical University of Lodz, 90-131 Lodz, Poland; 16 Department of Innovation, Merito University in Poznan, 61-895, Poznan, Poland

**Keywords:** public health, migrants, healthcare, GP

## Abstract

In 2020 it is estimated that 281 million people were international migrants. Migrants constitute a potentially vulnerable population in terms of facing discrimination, poor living and housing conditions, and insufficient access to healthcare services. Due to the armed conflict in Ukraine in 2022, almost 10 million people crossed the Polish border within a year of the outbreak of the conflict. The objective of this paper is to present the use of primary healthcare services by people migrating from Ukraine to Poland and identify the barriers in access to healthcare by this group of persons. This study used a qualitative research technique in the form of an expert interview using individual in-depth interviews (IDI). The study group consisted of professionally active primary healthcare providers (doctors, nurses, and facility managers) in Poland. Research was carried out in the areas regarding the availability of healthcare, the potential threats and challenges, and possible system solutions. The results showed that the most common cause for doctor’s appointments among migrants are respiratory infections, including COVID-19. Many cases were related to back pain, mainly resulting from the physical work of the patients. Additionally, some barriers to access and the provision of healthcare services for patients from Ukraine were identified. The majority (75%) of respondents indicated language as a significant barrier when providing services. Based on the study results, we recommend creating a dedicated website and telephone hotline for this group of persons as well as the use of traditional media to distribute information about access to healthcare services. It is also essential to focus on assistance for older people, since they may experience more difficulties with language and navigating the healthcare system.

## 1. Introduction

In 2020, it is estimated that 281 million people were international migrants [1]. Various studies suggest that migrants may experience discrimination, poor living conditions, and insufficient access to healthcare services [2,3]. This group is also burdened with chronic diseases, infectious diseases, and mental health problems due to stress and events that occurred before and during migration [4,5]. Social determinants have an impact on health outcomes and can be more dominant than the healthcare system; they could play a significant role in deepening inequalities between migrants and the host country population [6]. 

Within a year of the outbreak of the armed conflict on the territory of Ukraine in February 2022, almost 10 million people crossed the Polish border [7]. The peak of migration occurred in March 2022, when the arrival of approximately 2 million refugees was registered [8]. There are some crucial health aspects that need to be acknowledged in the context of migration, such as inequalities, social determinants of health, access to healthcare services, and legal frameworks. Migrants have various health needs, and the arrival of such large numbers poses a challenge to the entire health care system, in particular primary health care [5,6].

Primary healthcare in Poland covers various health services for insured citizens including the provision of disease prevention services and immunization, consultations on the treatment of diseases, laboratory, imaging, and non-imaging diagnostics (such as ECGs, X-rays, and ultrasounds), treatment in clinics and in the patient’s home, the prescription and management of medicine and medical devices, the issuing of referrals to specialist clinics, hospitals, etc. At the central level, the rights of Ukrainian citizens to health services in Poland are regulated primarily by the Act of 12 March 2022, on assistance to Ukrainian citizens in connection with the armed conflict in the territory of this country (Journal of Laws of 2022, item 583, as amended) and other legal acts [9]. In accordance with this act, Ukrainian citizens who came to Poland after 24 February 2022, can benefit from medical care on the same terms as Polish citizens.

According to research, migrants are exposed to unequal access to health care services compared to citizens of a given country, and it also happens that the health priorities of a given country and migrants are divergent, which makes it difficult to meet the expressed health needs of migrants [2,3,4]. The health status of patients is also important due to differences in the level of immunization or the scope of mandatory vaccinations. Vulnerable groups are disproportionately affected by health risks and barriers in access to health care. Particularly vulnerable groups in conflict-affected regions include people over 60, people with disabilities, children and young people, women and girls, and internally displaced people. The majority of migrants from Ukraine are women and children, which increases the demand for services addressed to this group of patients [10,11].

The objective of this article is to present how primary healthcare services are used by people migrating from Ukraine to Poland and the barriers in access to services in the ongoing migration crisis.

## 2. Materials and Methods

This research was conducted in the period of November–December 2023 and used a qualitative research technique in the form of individual in-depth interviews (IDI). The answer provided by each expert had equal weight and had the same importance for the conclusions reached in this research [12]. Information enabling the identification of individual respondents was not collected; therefore, the principle of anonymity has been maintained. The interviews were semi-structured, where the basis of the research tool was closed-ended questions, allowing for additional questions to be generated to clarify the statement. Research was carried out in problem areas regarding the availability of healthcare, potential threats and challenges, and possible system solutions. In the part regarding the assessment of possible organizational solutions, the basis was the report of the World Health Organization and Statistics Poland entitled: “Health of refugees from Ukraine in Poland 2022. A household survey and behavioral in-sights research.” The following recommendations were assessed: “Providing an interpreter with a knowledge of medical terminology in clinics”, “Providing step-by-step guidelines on how and where to get medical services, especially for children with special needs”, “Creating a telephone hotline to support making doctor’s appointments”, “Support training for employees of clinics that serve Ukrainians to encourage empathy, patience, clear communication and provision of full services”, and “Increasing opportunities for Ukrainians to enroll for health insurance”. To analyze the data collected during the IDI, statistical analysis methods were applied using Cohen’s Kappa agreement measures. A more detailed description of the performed analysis is presented beneath Table 1.

Twenty representatives of primary healthcare providers (POZ) took part in the research, including six doctors, six nurses, and eight representatives of facility management. Such a number allowed us to achieve a state of theoretical saturation based on the authors’ experience. The research involved professionally active representatives of primary healthcare providers (doctors, nurses, and facility managers). The term “professionally active” refers to those healthcare professionals who, at the time of this study, were working directly with patients in publicly funded healthcare facilities (as opposed to those working in the private sector or those who are licensed healthcare professionals but are employed elsewhere or retired). The inclusion criterion was contact with patients covered by the support system guaranteed by the Act of 12 March 2022 on assistance to citizens of Ukraine in connection with the armed conflict on the territory of this country (Journal of Laws 2022, item 583) [9], the Act of 23 March 2022 amending the Act on assistance to citizens of Ukraine in connection with the armed conflict on the territory of this country and the Act—Law on Higher Education and Science (Journal of Laws 2022, item 682) [13], the Act of 13 June 2003 on granting protection to foreigners on the territory of the Republic of Poland (Journal of Laws 2003, No. 128, item 1176) and other legal provisions [14]. This study was conducted in selected cities based on the analysis of data on the presence of the largest number of persons from Ukraine: Warsaw, the Tricity, Poznań, Kraków, Łódź, and Katowice [15]. According to Polish law, all publicly funded primary healthcare facilities should provide the same services, hence geographical differences were not taken into account in the analyses.

The level of agreement is determined in intervals according to the following principles:

- Very little agreement, values: 0.01 ÷ 0.20;
- Little agreement, values: 0.21 ÷ 0.40;
- Average agreement, values: 0.41 ÷ 0.60;
- Significant agreement, values: 0.61 ÷ 0.80;
- Perfect agreement, values 0.81 ÷ 1.00.

The table above presents the differences in the level of agreement between individual respondents, which indicates the diversity of answers. No clear trend in the agreement of answers was observed, e.g., in the professional group of doctors or nurses, which may be due to the fact that, in different facilities, the respondents had contact with different groups of patients, e.g., in terms of age or socio-economic status.

## 3. Results

### 3.1. Use of Services by Patients from Ukraine

**Figure 1:**
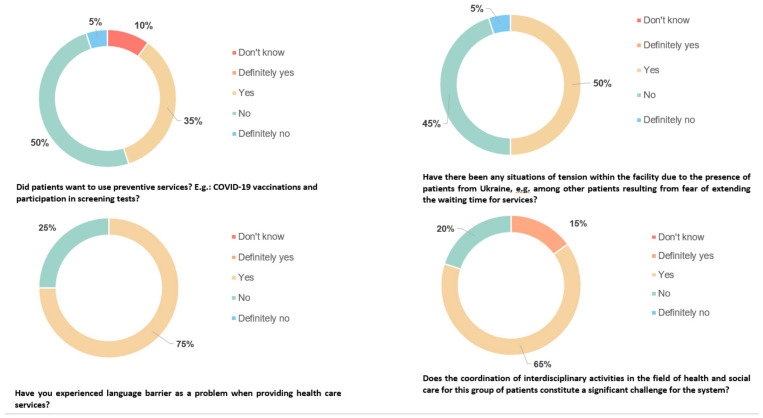
Challenges, barriers, and methods of using services by patients from Ukraine based on the authors’ research of healthcare providers.

The results of qualitative research of healthcare providers indicate several key reasons for using healthcare by patients from Ukraine. The most common cases are respiratory infections, including COVID-19 (U07.1, according to ICD-10). Many cases are related to back pain, mainly resulting from physical work (M40–M54, according to ICD-10). Taking into account the types of medical services, patients from Ukraine most often seek medical advice in order to have a prescription issued. Situations when they need diagnostics (e.g., an ultrasound examination or an X-ray) or referral to specialists (e.g., an orthopedist or an allergist) are slightly less common. It was noted that vaccinations, including those against COVID-19, are not very common among patients from Ukraine. According to the research results, 50% of respondents indicated that patients from Ukraine are not willing to use preventive services, including preventive vaccinations (Figure 1). The respondents pointed out the differences in the healthcare systems in Poland and Ukraine. There are cases where some medicines commonly available in Ukraine require a prescription in Poland, which can cause confusion among migrants. Moreover, Ukrainians are surprised and frustrated by the long waiting time to see a specialist. The respondents emphasized that patients from Ukraine were aware of their rights as patients and expected those services to be provided. The analysis of the results indicates the diverse health needs of migrants from Ukraine, but also some challenges related to integration into the Polish healthcare system.

The statements of representatives of primary healthcare providers show that the use of preventive services (e.g., vaccinations against COVID-19 and participation in screening tests) by patients from Ukraine depends on many factors. Attitudes towards vaccinations and other preventive activities seem to be based on individual beliefs, experiences, and needs. The respondents, who notice the willingness of Ukrainians to use preventive services, pointed out that some patients volunteer for vaccinations or tests, and some require a doctor’s persuasion (Figure 1). However, it should be emphasized that these patients usually follow the doctor’s recommendations and, after receiving a referral, use preventive services. It is also noted that adults seem to be less interested in vaccinating themselves than their children. Respondents who encounter Ukrainian reluctance to using preventive services indicated that some patients express concerns about vaccinations, especially against COVID-19. According to data, in 2021, approximately 20% of children in Ukraine were not fully vaccinated against the measles, and 13% were not fully vaccinated against polio [16,17]. Similarly, in the case of vaccinations against COVID-19, according to data from before February 2022, approximately 35% of the general population in Ukraine had received two doses of the vaccination, while in the rest of Europe it was approximately 65% of the population [18]. Despite doctors and nurses encouraging and presenting the benefits of vaccinations, they are not interested in using this type of services. This is due to, among other things, the belief that vaccinations are harmful or unnecessary because there are alternative methods of treatment. The lack of vaccinations is also partly due to the fact that employers do not impose an obligation on employees to get vaccinated, both against COVID-19 and influenza.

The respondents also pointed to problems with the use of services by migrants when the waiting time for a given service was too long. It occurred that more affluent patients from Ukraine resigned from services provided within the public system in favor of private appointments. However, in the case of patients with lower income, there were situations when, due to a lack of financial possibilities, newly employed people resigned from performing paid tests as part of occupational medicine (not every employer covers the costs of preliminary tests) or did not perform paid tests ordered by a doctor in a clinic. Some patients avoid using medical services until the situation forces them to do so. There were opinions that patients from Ukraine often ignore the early symptoms of the disease or cope on their own (e.g., using home remedies) and only visit a clinic when the disease is at an advanced stage.

Additionally, the respondents indicated tensions within medical facilities due to the provision of services to patients from Ukraine. Half of the respondents admitted that such situations occurred (Figure 1). Due to the migrants’ belief that they had priority in access to services, they expected that they would be seen out of turn. This caused frustration among other patients who felt a sense of injustice. The respondents pointed out that the growing number of patients from Ukraine affected the atmosphere in clinics, causing irritation among other patients. Medical staff had to perform mitigating interventions, explaining to Ukrainian patients that there were certain procedures that had to be followed. According to the special act, which entered into force on 12 March 2022, refugees and non-refugees from Ukraine have the right to medical services on the same terms as people insured in Poland. This means that regardless of nationality, every patient has the right to have his or her access to medical services in medical facilities determined by medical criteria, health condition, and place on the waiting list. Some respondents, however, pointed out that conflict situations caused by the demanding attitude of Ukrainian patients are becoming less frequent.

### 3.2. Barriers in Access/Provision of Services

An undoubted barrier to the access and provision of healthcare services for patients from Ukraine is the language barrier. The vast majority (75%) of respondents indicated language as a significant barrier when providing services (Figure 1). The statements of representatives of primary healthcare providers show that the inability to speak the language is a problem in communicating between medical staff and patients from Ukraine, which leads to difficulties in obtaining complete information about their health. The analysis indicates that the language barrier applies, in particular, to older people who have certain limitations in learning a new language. However, it was emphasized that this is not a rule, as it happens that people aged 25–35 also seem to experience problems with the Polish language. A lack of basic knowledge of English is also quite common, even among young people. The remaining respondents believe that the language barrier was not a problem when providing healthcare services. It was emphasized that, over time, patients from Ukraine, especially those staying in Poland for a long time, gradually master the Polish language. Younger people seem to learn Polish quickly and are able to use electronic translators. They want to learn the language quickly, which facilitates communication with medical staff. The younger generation, especially people who work and have contact with the Polish language on a daily basis, seem to be more engaged in the learning process. In the context of effective communication, support from Polish families who have accepted refugees from Ukraine is also relevant.

### 3.3. Challenges and Solutions for the Future

One of the elements of the performed analyses was to obtain information regarding the assessment of selected recommendations for systemic solutions developed by the World Health Organization and Statistics Poland. The respondents were asked to indicate the extent to which they agreed with them.

The recommendation “Providing an interpreter with knowledge of medical terminology in large medical facilities or clinics providing health care to a large number of patients from Ukraine under public financing” seems to raise varied opinions among the respondents. People who responded positively to the above recommendation justified it by the need to minimize the stress of Ukrainian patients and facilitate communication in urgent situations. Some respondents emphasized that the need for an interpreter may depend on many factors, such as the number of patients from Ukraine, the ability of medical staff to communicate in Ukrainian, or the availability of other communication methods, such as online translators. It was noticed that modern technologies can provide effective communication solutions. A good solution could be to provide system access to an online or telephone interpreter. In turn, opponents of this solution pointed primarily to the generation of additional costs related to the employment of an interpreter. Moreover, respondents indicated that employing an interpreter with knowledge of medical terminology could be justified only in exceptional situations, because the number of patients from Ukraine is not large enough to require employing an interpreter on a permanent basis. The presence of a doctor from Ukraine in the clinic may also be sufficient.

The recommendation “Providing detailed, easily accessible guidelines for both patients and providers on how and where to obtain medical services, especially for children with special needs” was positively received by most respondents. Supporters of this solution expressed the belief that its implementation would be beneficial not only for Ukrainians, but also for Poles, but the information provided must be presented in a simple and transparent way. The respondents indicated that a general understanding of medical procedures is important for all patients. Clear guidelines are also useful for medical staff, as they help eliminate ambiguities regarding the availability of services. The need to create a website where detailed information on medical services could be found has been mentioned. The respondents also suggested presenting information using leaflets and posters that would be publicly available and, at the same time, easy to notice and understand. Respondents emphasize that especially older people who do not use the Internet can use this traditional medium.

The recommendation “Creating a telephone hotline for this group of patients to facilitate making medical appointments” was met with mixed responses from the respondents. Some respondents seem to perceive it as beneficial, pointing out that such a hotline could play an intermediary role—both to contact clinics and provide information to patients. The respondents emphasize that the hotline should provide the necessary information regarding making medical appointments. They point to the helpful role of the hotline in providing detailed information and guiding patients through the process. Some respondents suggest that the situation is currently manageable, but, at the same time, they express a positive attitude towards a possible hotline, should one be established. In turn, opponents of the proposed solution suggest that there are no major difficulties in arranging medical appointments for patients from Ukraine. They emphasize that patients from Ukraine can communicate in Polish and are able to arrange appointments. According to some respondents, a better approach would be to improve overall access to medical services, instead of creating a separate hotline for a specific group of patients.

Representatives of primary health care providers participating in the research also indicated proposals for solutions that should be implemented in order to improve the functioning of the system at the level of primary health care for both patients and health care providers. These include the following:

1. The development of clear guidelines and recommendations for healthcare providers regarding the management of patients from Ukraine. The guidelines should include registration procedures, patient rights, and billing methods;
2. The creation of a hotline dedicated to the registration of patients from Ukraine, operated by a person who can speak both English and Ukrainian;
3. Increasing the time allocated for appointments to patients from Ukraine so that doctors can provide more complete care. This may be important especially in the context of complicated cases and communication problems;
4. The creation of bulletins, posters, and other information materials addressed to patients from Ukraine, which should precisely indicate where to go in specific situations and contain information on patient rights;
5. Introducing prior verification of appointments to minimize unjustified cases. This may include introducing a nominal upfront fee for appointments.

## 4. Discussion

People migrating to Poland from Ukraine have diverse health needs and vary in terms of education, income, and ability to navigate and use the Polish healthcare system. Moreover, there is a lack of further information on their health needs and health status. These findings are consistent with the results of other studies on this topic [19]. As shown in the authors’ research, migrants coming to Poland are rather reluctant to be vaccinated. This may be caused by several factors, including widespread doubt about the effectiveness and safety of vaccinations, partly exacerbated by social media campaigns that have served to spread disinformation about vaccinations and undermine public trust in authorities [20]. According to the World Health Organization, vaccine hesitancy means “a delay in accepting or refusing to accept safe vaccinations despite the availability of vaccination services” [21]. This is caused by a number of factors, including trust in the product, self-satisfaction, and comfort. Uncertainty about the safety of vaccinations is one of the factors that may cause reluctance towards vaccinations; it is often intensified by negative beliefs based on myths, e.g., about the negative impact of vaccinations on fertility or the spread of erroneous information regarding vaccinations. Due to the low level of immunization in Ukraine, an outbreak of a measles epidemic was recorded in 2017–2020, during which approximately 115,000 new cases were registered, making it one of the largest outbreaks in Europe [16]. This situation is particularly important in the context of providing healthcare services to patients from Ukraine in Poland. Taking into account the increasing popularity of anti-vaccination movements, it is important to strengthen positive attitudes towards vaccinations in Polish society, as well as among migrants.

The language barrier is another challenge related to providing services to people during the migration crisis [22,23,24]. In the study conducted by Pandey et al., language proficiency has been shown to significantly impact a patient’s ability to identify needed services, make appointments, and cooperate effectively with healthcare providers [25]. This research also showed that patients with language difficulties are less likely to actively use medical services. The study by Biesiada et al. emphasizes the role of primary health care in the process of providing services to migrants. This study also highlighted the importance of the language barrier as one of the main challenges in migrant health care [26]. The way healthcare is organized varies from country to country, and for newcomers, their understanding of the services provided in the host country depends largely on their ability to absorb information about them. People experiencing a language barrier may not know how to access various services in the system. The authors’ research showed that some primary healthcare providers notice the problem of lack of knowledge of the Polish language, especially among older Ukrainians, which makes it difficult to obtain complete information about their health and may lead to misunderstandings between patients and healthcare providers. Another study, by Jaeger et al., underlines that the language barrier is especially prominent in primary healthcare [27]. This paper also emphasizes that professional interpreter use is beneficial for both patients and physicians [28]. Similar results were also obtained in other studies on the communication aspects of healthcare services [29,30].

### Limitations

The main objective of this research was to assess the answers of the healthcare system and healthcare providers to the emerging migration crisis. This research used the qualitative technique of individual in-depth interviews, while other studies on migration mainly used the technique of quantitative research or literature review. Another advantage of this research is the examination of this phenomenon from the point of view of healthcare providers in order to fully determine the challenges faced by the Polish health care system. Due to the qualitative research technique used, this research also has some limitations—the results are more subjective, they cannot be directly extrapolated to the entire population, and the phenomenon of recall bias is also observed.

## 5. Conclusions

The inflow of migrants to Poland caused by the armed conflict in Ukraine has burdened the health care system in Poland, especially at the level of primary health care. The Polish health care system is different from the one in Ukraine, which may pose a challenge in using health services, especially among older patients. Due to the diverse health needs and socio-demographic characteristics of migrants, we recommend creating a dedicated website and telephone hotline for this group of patients as well as using the traditional media (leaflets and posters) to disseminate information about healthcare services. Activities aimed at this group of patients should be strengthened to promote preventive services, particularly vaccinations. Moreover, additional assistance for older patients should be provided since they may experience more difficulties with a language barrier and navigating the healthcare system. The role of employers in insuring employees from Ukraine should also be emphasized.

## Figures and Tables

**Table 1:** The results of the analysis of Cohen’s Kappa agreement measures.

TYPE	L-Ł	P-W	P-T	A-Ł	A-T	A-Ł	L-Ł	L-T	L-Ł	L-Ł	P-T	P-K	P-Ł	A-P	A-K	A-P	L-W	A-W	A-Ka	P-W
**L-Ł**	-	0.35	0.15	0.11	0.04	0.22	0.05	0.19	0.11	−0.01	0.50	0.27	0.23	0.12	0.10	0.34	0.13	0.29	−0.17	0.09
**P-W**	0.35	-	−0.10	−0.29	0.18	−0.34	0.09	0.07	0.32	0.05	0.36	−0.22	−0.1	0.00	−0.18	0.23	0.12	0.17	−0.26	0.01
**P-T**	0.15	−0.10	-	0.55	0.22	0.09	0.15	0.35	−0.28	0.24	−0.05	0.45	0.33	−0.07	0.18	0.17	0.05	0.22	0.28	−0.02
**A-Ł**	0.11	−0.29	0.55	-	0.05	0.35	0.01	0.22	−0.13	0.07	−0.02	0.25	0.38	0.12	0.25	0.03	−0.19	0.33	0.25	0.05
**A-T**	0.04	0.18	0.22	0.05	-	−0.25	0.33	0.37	−0.04	0.73	−0.17	−0.28	0.27	0.22	−0.02	0.26	0.18	−0.16	−0.09	0.13
**A-Ł**	0.22	−0.34	0.09	0.35	−0.25	-	−0.06	0.06	0.04	−0.17	−0.04	0.08	0.06	0.09	−0.02	−0.3	0.01	0.41	0.24	−0.02
**L-Ł**	0.05	0.09	0.15	0.01	0.33	−0.06	-	0.38	0.06	0.22	0.33	0.19	0.22	0.28	0.19	0.38	0.32	−0.10	−0.02	0.17
**L-T**	0.19	0.07	0.35	0.22	0.37	0.06	0.38	-	−0.11	0.50	0.27	0.11	0.61	0.10	0.12	0.31	0.01	0.14	−0.32	0.29
**L-Ł**	0.11	0.32	−0.28	−0.13	−0.04	0.04	0.06	−0.11	-	−0.09	0.32	−0.12	−0.21	0.09	−0.06	0.04	0.01	0.23	−0.08	−0.06
**L-Ł**	−0.01	0.05	0.24	0.07	0.73	−0.17	0.22	0.5	−0.09	-	0.17	0.03	0.43	0.28	0.04	0.38	0.19	−0.22	0.02	0.32
**P-T**	0.5	0.36	−0.05	−0.02	−0.17	−0.04	0.33	0.27	0.32	0.17	-	0.20	0.07	0.2	0.21	0.22	0.08	0.14	−0.28	0.28
**P-K**	0.27	−0.22	0.45	0.25	−0.28	0.08	0.19	0.11	−0.12	0.03	0.20	-	0.05	−0.06	0.15	0.32	0.16	0.02	0.46	−0.18
**P-Ł**	0.23	−0.10	0.33	0.38	0.27	0.06	0.22	0.61	−0.21	0.43	0.07	0.05	-	−0.13	0.29	0.37	−0.02	0.25	−0.25	0.62
**A-P**	0.12	0.00	−0.07	0.12	0.22	0.09	0.28	0.10	0.09	0.28	0.20	−0.06	−0.13	-	0.35	0.37	0.42	0.03	0.12	0.10
**A-K**	0.10	−0.18	0.18	0.25	−0.02	−0.02	0.19	0.12	−0.06	0.04	0.21	0.15	0.29	0.35	-	0.50	0.15	0.30	0.25	0.42
**A-P**	0.34	0.23	0.17	0.03	0.26	−0.30	0.38	0.31	0.04	0.38	0.22	0.32	0.37	0.37	0.50	-	0.28	0.15	0.22	0.31
**L-W**	0.13	0.12	0.05	−0.19	0.18	0.01	0.32	0.01	0.01	0.19	0.08	0.16	−0.02	0.42	0.15	0.28	-	0.05	0.19	0.02
**A-W**	0.29	0.17	0.22	0.33	−0.16	0.41	−0.10	0.14	0.23	−0.22	0.14	0.02	0.25	0.03	0.3	0.15	0.05	-	0.05	0.11
**A-Ka**	−0.17	−0.26	0.28	0.25	−0.09	0.24	−0.02	−0.32	−0.08	0.02	−0.28	0.46	−0.25	0.12	0.25	0.22	0.19	0.05	-	−0.20
**P-W**	0.09	0.01	−0.02	0.05	0.13	−0.02	0.17	0.29	−0.06	0.32	0.28	−0.18	0.62	0.10	0.42	0.31	0.02	0.11	−0.20	-

Explanation of symbols from the table (e.g., L-Ł means Doctor-Łódź): First symbol: L—Doctor; P—Nurse; and A—Administration—Facility Manager; Second symbol: W—Warsaw; T—Tricity; P—Poznań; K—Kraków; Ł—Łódź; and Ka—Katowice. The table above presents the results of analysis of Cohen’s Kappa agreement measures (Table 1). The interpretation of the results of agreement measures ranges from 0 to 1, where 0 means no agreement between two concepts and 1 means perfect agreement (Table 1). Using statistical methods, it is possible to mathematically measure qualitative variables in a given case—the respondents’ answers. Cohen’s Kappa agreement measure measures the consistency of ratings of people assessing the same object on the same scale, expressing the likelihood of correct classification using a validated method compared to a reference method or retest. Higher Kappas mean greater consistency in measurements. A value of 1 indicates perfect agreement. A value of 0 means that the agreement is no better than random. The colours differentiate levels of agreement between respondents, where red indicates low level of agreement, white indicates levels close to 0 and blue indicates high level of agreement.

## Data Availability

The original data collected to conduct this study might be available from the authors.

## References

[1] WHO—Essential Knowledge—Health and Migration. https://www.who.int/tools/refugee-and-migrant-health-toolkit/essential-knowledge-health-and-migration.

[2] Di Napoli A., Petrelli A., Rossi A., Mirisola C., Rosano A., Rosano A. (2018). Access to Medical Examination for Primary Prevention Among Migrants. Access to Primary Care and Preventative Health Services of Migrants.

[3] Szaflarski M., Bauldry S. (2019). The Effects of Perceived Discrimination on Immigrant and Refugee Physical and Mental Health. Adv. Med. Sociol..

[4] Debesay J., Nortvedt L., Langhammer B. (2022). Social Inequalities and Health among Older Immigrant Women in the Nordic Countries: An Integrative Review. SAGE Open Nurs..

[5] Agyemang C., van den Born B.-J. (2019). Non-communicable diseases in migrants: An expert review. J. Travel Med..

[6] Zhang C.X., Boukari Y., Pathak N., Mathur R., Katikireddi S.V., Patel P., Campos-Matos I., Lewer D., Nguyen V., Hugenholtz G.C.G. (2022). Migrants’ primary care utilisation before and during the COVID-19 pandemic in England: An interrupted time series analysis. Lancet Reg. Health Eur..

[7] United Nations High Commissioner for Refugees (UNHCR) Operational Data Portal Ukraine Refugee Situation. Continually Updated..

[8] National Headquarters of the Border Guard Statistics. https://www.strazgraniczna.pl/pl/granica/statystyki-sg/2206,Statystyki-SG.html.

[9] Act of 12 March 2022 on Assistance to Citizens of Ukraine in Connection with an Armed Conflict on the Territory of This Country. https://isap.sejm.gov.pl/isap.nsf/DocDetails.xsp?id=WDU20220000583.

[10] Malmusi D., Drbohlav D., Dzúrová D., Palència L., Borrell C. (2014). Inequalities in healthcare access by type of visa in a context of restrictive health insurance policy: The case of Ukrainians in Czechia. Int. J. Public Health.

[11] Ukraine: Public Health Situation Analysis (PHSA)-Long-Form (Last Update: July 2022). https://reliefweb.int/report/ukraine/ukraine-public-health-situation-analysis-phsa-long-form-last-update-july-2022-enuk.

[12] Dicicco-Bloom B., Crabtree B.F. (2006). The qualitative research interview. Med. Educ..

[13] Act of 23 March 2022: Amending the Act on Assistance to Citizens of Ukraine in Connection with an Armed Conflict on the Territory of This Country and the Act–Law on Higher Education and Science. https://isap.sejm.gov.pl/isap.nsf/DocDetails.xsp?id=WDU20220000682.

[14] Act of 13 June 2003 on Granting Protection to Foreigners in the Territory of the Republic of Poland. https://isap.sejm.gov.pl/isap.nsf/DocDetails.xsp?id=wdu20031281176.

[15] https://businessinsider.com.pl/gospodarka/te-polskie-miasta-najbardziej-upodobali-sobie-uchodzcy-z-ukrainy/2l7epqs.

[16] Health Cluster Ukraine (2022). Ukraine Public Health Situation Analysis (PHSA)—Short-Form. https://healthcluster.who.int/publications/m/item/ukraine-public-health-situation-analysis-(phsa)---short-form.

[17] Hill M., Vanderslott S., Volokha A., Pollard A.J. (2022). Addressing vaccine inequities among Ukrainian refugees. Lancet Infect. Dis..

[18] Our World in Data COVID-19 Data Explorer. https://ourworldindata.org/coronavirus.

[19] Healthcare Needs among Refugees from Ukraine Arriving in Norway during 2022. https://fhi.brage.unit.no/fhi-xmlui/handle/11250/3086534.

[20] Broniatowski D.A., Jamison A.M., Qi S., AlKulaib L., Chen T., Benton A., Quinn S.C., Dredze M. (2018). Weaponized health communication: Twitter bots and russian trolls amplify the vaccine debate. Am. J. Public Health.

[21] Vaccine Hesitancy: A Growing Challenge for Immunization Programmes. https://www.who.int/news/item/18-08-2015-vaccine-hesitancy-a-growing-challenge-for-immunization-programmes.

[22] El Arab R.A., Urbanavice R., Jakavonyte-Akstiniene A., Skvarcevskaja M., Austys D., Mateos J.T., Briones-Vozmediano E., Rubinat-Arnaldo E., Istomina N. (2023). Health and social needs of asylum seekers and Ukrainian refugees in Lithuania: A mixed-method protocol. Front. Public Health.

[23] Al Shamsi H., Almutairi A.G., Al Mashrafi S., Al K.T. (2020). Implications of language barriers for healthcare: A systematic review. Oman Med. J..

[24] A Mile in their Shoes: Understanding Health-Care Journeys of Refugees and Asylum Seekers in the UK. https://www.helenbamber.org/resources/research/mile-their-shoes-understanding-health-care-journeys-refugees-and-asylum-seekers.

[25] Pandey M., Maina R.G., Amoyaw J., Li Y., Kamrul R., Michaels C.R., Maroof R. (2021). Impacts of English language proficiency on healthcare access use outcomes among immigrants: A qualitative study. BMCHealth Serv. Res..

[26] Biesiada A., Mastalerz-Migas A., Babicki M. (2023). Response to provide key health services to Ukrainian refugees: The overview and implementation studies. Soc. Sci. Med..

[27] Jaeger F.N., Pellaud N., Laville B., Klauser P. (2019). The migration-related language barrier and professional interpreter use in primary health care in Switzerland. BMC Health Serv. Res..

[28] Puthoopparambil S.J., Phelan M., MacFarlane A. (2021). Migrant health and language barriers: Uncovering macro level influences on the implementation of trained interpreters in healthcare settings. Health Policy.

[29] Rolke K., Walter J., Weckbecker K., Münster E., Tillmann J. (2024). Identifying gaps in healthcare: A qualitative study of Ukrainian refugee experiences in the German system, uncovering differences, information and support needs. BMC Health Serv. Res..

[30] Understanding Health-Seeking Behaviors and Barriers to Healthcare Access among Ukrainian Migrant Women Working in the Domestic Sector in Warsaw, Poland. https://www.econstor.eu/handle/10419/231799.

